# Impact of decentralized management on sickness absence in hospitals: a two-wave cohort study of frontline managers in Danish hospital wards

**DOI:** 10.1186/s12913-024-11234-2

**Published:** 2024-07-16

**Authors:** Thim Prætorius, Thomas Clausen, Ann Dyreborg Larsen, Jonas Kirchheiner Rasmussen, Lykke Margot Ricard, Peter Hasle

**Affiliations:** 1 Steno Diabetes Center Aarhus, Aarhus University Hospital, Aarhus, Denmark; 2 National Research Center for the Working Environment, Copenhagen, Denmark; 3 Department of Technology and Innovation, University of Southern Denmark, Odense, Denmark

**Keywords:** Decentralised decision authority, Cross-functional decision authority, Decision making, Sickness absence, Hospitals, Frontline managers

## Abstract

**Background:**

This study explores the impact of decentralized management on the sickness absence among healthcare professionals. Sickness absence is a reliable indicator of employees’ wellbeing and it is linked to management quality. However, the influence of decentralized management on sickness absence has not been adequately studied.

**Methods:**

The research design combined a two-wave, web-survey of frontline managers in two Danish university hospitals with administrative data on sickness absence at the ward-level. The first and second wave included data from 163165 and 137 frontline managers linked to 121 wards and 108 wards. Data was analysed using an ordinal logistic regression model.

**Results:**

Wards where frontline managers had the highest level of decentralised decision authority compared to none showed lower odds of ward-level sickness absence (OR_crude_: 0.20, 95% CI: 0.05–0.87). A very high extent of cross-functional decision authority showed lower odds of sickness absence (OR_crude_: 0.08, 95% CI: 0.01–0.49). Overall, the results showed a clear data trend, although not all results were statistically significant.

**Conclusion:**

Higher levels of decentralized management in wards were positively associated with lower risks of sickness absence in hospital wards. The study supports future research on how to empower decision autonomy at the frontline level of management.

**Supplementary Information:**

The online version contains supplementary material available at 10.1186/s12913-024-11234-2.

## Introduction

### Background

Sickness absence among healthcare staff is high on the political agenda in the OECD countries. More than ever, public hospitals face high care demands and with a shortage of healthcare professionals there is an urgent need for developing knowledge on how to manage and organize healthcare work to avoid burnout, absenteeism, and stabilize retention rates [1–4]. This article contributes to this discussion by studying the impact of decentralizing management on ward-level sickness absence. Decentralization to frontline managers in hospitals is a particularly important level to zoom in on because managers at the frontline are directly involved in helping work teams accomplish collective objectives, and the way managers manage is linked to the psychosocial work environment and well‑being of healthcare professionals [5–10], impacting the service performance of hospitals [11, 12]. Managers who give frontline workers adequate resources and encouragement are also found to strengthen workers' skill sets who in turn make better decisions [13].

This study adopts the perspective that empowered frontline managers are fundamental to hospital performance [14–16]. To explore this impact further, studies into enabling management structures [17, 18] point to two compelling management aspects related to decentralization. First, delegating decentralization of decision-making authority to frontline managers [19] is hypothesized to improve decision-making and collaboration at the frontline of care delivery [20]. Second, managers with cross-functional decision authority can span and connect across occupational boundaries [21] and is considered a mechanism for bridging healthcare professionals and units [22].

### Aim and contribution

The overall purpose of this explorative study is to evaluate the association between decentralized management in hospitals and sickness absence among healthcare professionals. We break the concept of decentralized management into vertical decision authority (who make decisions about what in the hospital) [19] and cross-functional decision authority (who make decisions outside their own occupation in the hospital) [17]. By investigating these two aspects of the managerial role, we analyse whether a) the level of decentral decision authority and b) the ability of managers to coordinate task completion across different job functions is associated with employee-well-being. These two management aspects have not been adequately considered nor linked to how it can improve the work environment in hospitals [23] and lead to a sustainable psychosocial work environment [24].

The contribution of the present study is threefold. First, by deploying an innovative study design using self-reported questionnaire data from line-managers combined with register-based information on sickness absence at the ward-level, we provide novel evidence on the association between leadership behaviour and employee well-being and heed the call for performing rigorous empirical research on the impact of management at a time where healthcare systems reorganize to meet changing demographics and patient demands [12]. Compared to previous studies primarily surveying nurse managers, our study sample consists of nurses and physicians who jointly undertake the role of frontline managers, exemplifying a recent trend in clinical leadership models [25]. Second, by investigating decentralized decision authority and cross-functional decision authority, we operationalize important elements of complex work organizations, such as hospitals, where the need for agile decision making processes are important to facilitate efficient and high-quality delivery of health care services involving employees with different professional backgrounds. The study differs from previous studies on hospital managers focusing on performance management [8] or specific leadership styles [12] such as distributed [14, 16] or relation-oriented leadership [5, 26]. Third, we contribute insights complementing research on psychosocial working conditions (e.g., high workload and low levels of social capital, job control, and organizational justice) found to be predictors of sickness absence [27–31]. We use sickness absence as an outcome measure because it may be considered an objective and reliable indicator of employees’ well-being and their work environment [7].

### Hypothesis development

Centralization refers to a situation where decision-making power rests with a central person or team in the center or at the top of the organizational hierarchy [32]. In contrast, decentralization means that the decision-making power is delegated to frontline managers or employees within an organization, and organizations do it because of the potential it has for improving operations and task completion [33, 34]. A high level of decentralization allows employees to make decisions on their own and empower their autonomy. Organizations delegate decision authority because there is a limit to how many people a manager can effectively manage, and delegation can improve decision quality, economise on managerial attention, and facilitate employee initiative. From the viewpoint of a manager, delegation also means losing control over delegated decisions that for some can be challenging to deal with [19, 35, 36]. Moving decision authority to the frontline manager in hospitals enhance the room for exercising supportive and flexible leadership behaviours that can meet employees' specific needs and situational demands [14, 16]. Delegating decision-making to the frontline manager also enhances a more enabling management structure by offering employees access to information, resources, and opportunities to influence decisions [1]. Employees are more likely to feel empowered with adequate discretionary power (needed as to do their job) with a positive effect on the well-being of employees working at the frontline. Decentralisation of decision authority is also attributed to a reduction in the risk of numbers of sick days due to a decrease in job burnout and increase in job satisfaction [24, 29]. We hypothesize that delegation of decision authority may be associated with lower levels of sickness absence at the ward level for several reasons. First, delegation of decision authority may boost job resources – e.g. job autonomy and supportive leadership behaviours – and the availability of these job resources may enhance the possibilities of health care workers to deal with the job tasks while simultaneously supporting the work-related well-being of health care workers through higher levels of work engagement and lower levels of burnout [37]. Second, following the meta-analysis from Miraglia and Johns [38], higher levels of leadership support and job control are associated with higher levels of job satisfaction and lower levels of sickness absence. Our first hypothesis can therefore be formulated as follows:

H1: Decentralization of decision authority in hospitals is associated with a reduced risk of sickness absence at the ward-level.

Since hospitals are characterized by strong professional cultures and high specialization, many contextual factors work against managing across organizational and occupational boundaries [39, 40]. However, increasingly complex care processes and patients’ need for integrated care makes it critical that frontline managers in hospitals are capable of supporting collaboration across occupational boundaries [41, 42]. This development emphasizes a need for managers who can bridge silos and create linkages between occupations and groups to move ideas, information, people and resources to where they are needed [17]. A decision-making perspective on the phenomenon of managing across occupational boundaries can be captured by the concept of cross-functional decision authority [43] concerned with the degree to which a manager in a hospital makes decisions outside their own occupation [12, 21, 44]. At the level of frontline management, cross-functional decision authority is hypothesised to support task completion and empower employees by providing access to supervision, information and resources across areas of specialisations [1], which is expected to be have a positive effect on the level of sickness absence in their wards [5, 7, 27]. Accordingly, we expect that higher levels of cross-functional decision authority may enhance the social capital at the ward-level by facilitating collaboration between different professional groups and strengthening the social capital in the work-group. Higher levels social capital at the ward-levels constitutes a job resource [45] that is associated with a decreased risk of sickness absence [27]. A meta-analysis [38] also indicates that social capital in the workplace (i.e., support from co-workers and supervisors) are indirectly associated with sickness absence and that the association is mediated by job satisfaction. Our second hypothesis can thus be formulated as:

H2: Cross-functional decision authority in hospitals is associated with a reduced risk of sickness absence at the ward-level.

## Methods

### Study setting

The study was carried out in two Danish university hospitals. Denmark has a universal, decentralized health system, in which the national government provides block grants from tax revenues to the regions, which operate hospitals, and to local authorities that, e.g., deliver prevention and rehabilitation services. Hospitals in Denmark are mainly public, paid through global budgets and case-based payments. Hospital physicians and nurses are salaried and employed by regional hospitals. All residents are entitled to publicly financed care, including largely free primary, specialist, hospital, mental health, preventive, and long-term care services. In Denmark, the hospital sector is among the work sectors with the highest prevalence of sickness absence with a level of 5.1 percent of the total working time. In 2019, assistant nurses and nurses respectively had the highest and fifth highest prevalence of sickness absence among all professional groups [46]. In Denmark, employees are eligible for sickness absence benefits if they cannot work due to sickness. Employees in hospitals owned by the Regional level of government are entitled to full wages while sickness absent and the employer is reimbursed by the Municipal level of government after 30 days of sickness absence.

### Study design and data collection

The target population for the survey was frontline managers with staff responsibility because they manage the wards where healthcare professionals provide patient care. The research design consisted of a two-wave web-survey that was developed for this study using insights from management theory [12, 33, 44, 47] (see Supplementary file 1). Survey data was collected in two Danish university hospitals in the Capital Region of Denmark (anonymized as City Hospital 1 and City Hospital 2). Based on a history of collaborating with the study team on research projects, the executive management team in the two hospitals granted access to collect the survey data. To identify our target group, we obtained an administrative list of managing physicians and nurse managers from the Capital Region of Denmark. In collaboration with the two participating hospitals, we identified all managers from units directly providing care: acute, elective, a combination or other. The final list was validated by the human resource departments to ensure accuracy. Some wards were managed by a team consisting of a managing physician and nurse manager, meaning that the number of managers participating in the survey can exceed the number of wards. Data were collected from frontline managers by use of their personal work email.

The first wave of data collection took place from November 2018 to January 2019 and the second wave from November 2019 to January 2020. To encourage participation, a member of the executive hospital management signed the survey invitation, which was distributed electronically using SurveyXact. To increase the response rate, three e-mail reminders were sent out in each wave. Each invitation letter contained information about the study, a unique survey link, and participation information about data protection and anonymity. Participation was voluntary. In the first wave of data collection, 369 frontline managers received the questionnaire with a response rate of 58.3% (165 frontline managers). In the second wave of data collection, 310 frontline managers received the questionnaire with a response rate of 58.4% (137 frontline managers). Across the two waves, the same 125 frontline managers responded to the questionnaire in both. The sample is tied to 121 wards with 3,680 employees in the first wave and 108 wards with 3,331 employees in wave two. Due to the low number of observations on the independent variables, we analysed data from the two waves independently to identify similarities in the patterns of the results. The employees in the participating wards were not invited to participate in the survey.

By combining the survey data with administrative data on sickness absence, the wards represent the unit of analysis. The first wave of the survey was merged with register-based data on sickness absence in the 121 wards from the period of January 2019 to December 2019. The second wave of the survey was merged with register based data on sickness absence in the 108 wards from the period of January 2020 to December 2020. Data was managed in accordance with GDPR guidelines.

### Measures

The construct ‘Decentralization of decision authority’ included five items taken from previously used questionnaires [33, 47] and reworded to fit the context of hospital management: who has decision authority with regard to: 1) prioritizing projects at the department, 2) collaboration with other departments at the hospital, 3) decisions with regard to quality control, 4) significant changes in patient service, and 5) significant changes in departmental routines. The response categories were as follows: the decision authority lies with 1) employees under my leadership, 2) myself (and co-managers), 3) my immediate supervisor, 4) the top management. We constructed an additive index on decentralization of decision authority by counting the number of items where the manager responds that either the manager or the employees under his/her supervision had the decision authority. The additive index ranged from 0–5, where 0 refers to no local decision authority and the higher the number, the greater the local decision authority. The internal consistency of the measures was assessed via Cronbach alphas and lay above the acceptable threshold of 0.70.

We measured the degree of exercising ‘cross-functional decision authority’ – i.e., make decisions across functions [12, 44] – with the question ‘to what extent do you on a daily basis exercise management for other occupational groups than your own’. It was measured using a 5-point Likert scale ranging from ‘not at all’ to ‘to a very great extent’. This measure drew on the concept of boundary-spanning leadership, and it was self-developed to meet the research aim for which it was not possible to find a suitable and pre-used questionnaire.

The survey data came from a larger questionnaire covering: respondent characteristics (e.g., profession and management experience); management responsibilities (e.g., operations); unit characteristics (e.g., type and size); management team (e.g., composition and span of control) and coordination mechanisms (e.g., plans, rules and roles).

The data on the outcome measure of sickness absence at the ward-level was obtained from administrative databases from the Capital Region of Denmark known to be highly reliable and accurate and have limited issues of biased or missing data [48]. To handle the different number of employees per ward, the annual number of absent days was calculated against the expected annual working days at each ward, and finally categorized the ordinal variable as: 1 = less than 3% sickness absence, 2 = 3–6% sickness absence, and 3 = more than 6% sickness absence. If we assume that the expected annual working days/year are approx. 220, then less than 3% reflect 6,5 days of sickness absence per year. By categorising sickness absence as an ordinal variable, it was possible to use ordinal logistic regression analysis, which allows for greater contrast in sickness absence than can be obtained in standard logistic regression models with only two outcomes. Further, this type of analysis does not assume normality, linearity or homoscedasticity, which are seldom obtainable with data on sickness absence [49].

### Covariates

Manager and hospital ward characteristics were included as covariates because of their potential influence on leadership, the work environment or sickness absence [50]. All covariates were included a priori, and then tested in different models to see if they change the results. The following covariates was utilised from the surveys: Manager characteristics: sex (female, male, other, do not want to inform); job category (doctor, nurse, other); management training (yes/no); years of management experience (numerical); number of employees working under their management (1 = 0–9 employees, 2 = 10–14 employees, 3 = 15–24 employees, 4 = more than 24 employees). Hospital ward characteristic: performing acute care tasks (yes/no); and hospital (1 or 2).

### Statistical analysis

Descriptive analyses assessed the hospital and ward-level according to characteristics of the hospitals and frontline managers. The association between each of the two structural aspects of cross-functional decision authority and decentralization of decision authority and sickness absence was analysed using multinomial logistic regression models. Because the ordinal response categories have an order, we refer to this as ordinal logistic regression analysis [51]. This analytical approach models the relationship between an ordinal response variable, in this case sickness absence, and one or more explanatory variables here the ‘cross-functional decision authority' variable based on a 5-point Likert scale, and the ‘decentralization of decision authority' on an ordinal scale. The ordinal logistic regression analyses were performed using the PROC GENMOD procedure in SAS version 9.4 (SAS Institute Inc., Cary, NC, USA).

To study the two hypotheses, we analysed the associations between the following items and the sickness absence data that we were granted access to:

- The additive index on decentralized decision authority and sickness absence measured as annual sickness absence at ward-level in the year after the start of data collection.
- Decentralized decision authority single items and sickness absence measured as annual sickness absence at ward-level in the year after the start of data collection to test if any of the single item are more important than others.
- Cross-functional decision authority single item and sickness absence measured as annual absence at ward-level in the year after the start of data collection.

We made the following adjustment in a total of four models: hospital (model 1); model 1 plus managers’ job category, management training and years of management experience (model 2); model 2 plus span of control (model 3); model 3 plus acute tasks (model 4).

The analyses were stratified by survey wave (1 or 2). The two waves were treated separately due to differences in both questionnaire responses and ward-level sickness absence. Results from the same department were treated as repeated measurements (repeated subject in the SAS procedure PROC GENMOD), e.g., if the management is shared between a managing physician and a nurse manager. The overall level of statistical significance was set at 0.05.

The interpretation of the ordinal regression model is: when the sickness absence scale is demarcated as 1 (= less than 3% absence per year), 2 (= 3–6%absence per year) and 3 (= more than 6% absence per year) then an OR of 0.40 means that the odds of “3” vs “1 or 2” are 60% lower among the exposed group than in the reference group. It also means that the odds of “2” vs “1” are 60% lower among the exposed group than the reference group. The assumption of the cumulative logistic regression is that the odds ratio for being in category “3” vs “1 or 2” is the same as the odds ratio for being in category “2” vs. “1”..

### Validity and reliability

Prior to the first wave of the survey and to test its face validity, the questionnaire was evaluated by and revised according to the inputs from 10 frontline managers working in the two hospitals. Because the data on the decentralization of decision authority and cross-functional decision authority have been gathered as self-reported survey data, they could be subject to recall-bias, but it is difficult to obtain information on these variables in any other way. Common method bias is a risk when correlating data in the same survey or between latent constructs based on self-reported items and believed leadership qualities. However, since we combine the self-reported survey data (i.e., our predictor variables) with administrative data (i.e., sickness absence as the outcome variable at the ward-level), these risks of common method bias have been mitigated [52].

## Results

Table 1 shows the descriptive statistics for the variables studied. The table is stratified by data wave and hospital and shows that around 80% of the study population of frontline managers were women (first wave 82% (*n* = 134) and second wave 79% (*n* = 108)). The most predominant job type was nurse (64% (*n* = 105) and 62% (*n* = 85)) followed by physician (25% (*n* = 41) and 28% (*n* = 38)). On average, the managers had around twelve years of management experience (11years (*n* = 165) and 12 years (*n* = 137)) and a high percentage had received management training (89% (*n* = 147) and 93% (*n* = 127)). The span of control varied from zero to more than 24 persons in both waves. Of the wards in the first and second data wave, 18 and 16% performed acute tasks, 26 and 24% were primarily elective, and the remaining 58% performed combinations of acute and elective tasks, or other care tasks.

**Table 1:** Descriptive statistics for main study variables for the respondents in the first and second survey wave

		1st data wave							2nd data wave				
		City Hospital 1		City Hospital 2		Total		City Hospital 1		City Hospital 2			Total
		*N*	%/mean (*SD*)	*N*	%/mean (*SD*)	*N*	%/mean (*SD*)	*N*	%/mean (*SD*)	*N*	%/mean (*SD*)	*N*	%/mean (*SD*)
Frontline managers		93	56.36	72	43.64	165	100	81	59.12	56	40.88	137	100
Ward-level sickness absence %/year													
< 3%		18	19.35	13	18.06	31	18.79	21	25.93	12	21.43	33	24.09
3–6%		56	60.22	37	51.39	93	56.36	38	46.91	12	21.43	50	36.50
> 6%		19	20.43	22	30.56	41	24.85	22	27.16	32	57.14	54	39.42
Gender													
Woman		81	87.10	53	74.65	134	81.71	66	81.48	42	75.00	108	78.83
Man		12	12.90	18	25.35	30	18.29	15	18.52	14	25.00	29	21.17
Job type													
Nurse		68	73.12	37	51.39	105	63.64	54	66.67	31	55.36	85	62.04
Physician		12	12.90	29	40.28	41	24.85	17	20.99	21	37.50	38	27.74
Other		13	13.98	6	8.33	19	11.52	10	12.35	4	7.14	14	10.22
Management experience (years)		93	12.06 (8.37)	72	10.47 (8.24)	165	11.37 (8.32)	81	13.00 (8.75)	56	11.09 (6.99)	137	12.22 (8.11)
Management training													
Yes		88	94.62	59	81.94	147	89.09	78	96.30	49	87.50	127	92.70
No		5	5.38	13	18.06	18	10.91	3	3.70	7	12.50	10	7.30
Span of control (# employees)													
0–9		15	16.13	19	26.39	34	20.61	20	24.69	20	35.71	40	29.20
10–14		25	26.88	22	30.56	47	28.48	16	19.75	12	21.34	28	20.44
15–24		24	25.81	15	20.83	39	23.64	22	27.16	12	21.43	34	24.82
> 24		29	31.18	16	22.22	45	27.27	23	28.40	12	21.43	35	25.55
Care task type													
Acute		10	10.75	20	27.78	30	18.18	8	9.88	14	25.00	22	16.06
Elective		22	23.66	17	23.61	39	23.64	19	23.46	17	20.36	36	26.28
Both or others		61	65.59	35	48.61	96	58.18	54	66.67	25	44.64	79	57.66

Table 2 shows the ordinal logistic regression analyses on decentralized decision authority measured as an additive index and ward-level sickness absence. The analyses are stratified by data wave and are in four models adjusted for hospital, manager's job category (doctor, nurse or other), management training, management tenure, span of control, and providing acute tasks. The results indicate a tendency in both waves, namely that higher levels of decision authority are associated with lower risk of sickness absence at the ward-level. However, only few of the reported odds ratios in Table 2 are statistically significant and none of the overall tests are statistically significant. Stepwise adjustment for the included covariates (Model 1- 4) did not substantially change the risk estimates.

**Table 2:** Results from multinomial logistic regression analysis of the association between the additive index on decentralized decision authority and ward-level sickness absence

		Crude			Model 1^A^			Model 2^B^			Model 3^C^			Model 4^D^		
		OR	95%-CI	P^E^	OR	95%-CI	P^E^	OR	95%-CI	P^E^	OR	95%-CI	P^E^	OR	95%-CI	P^E^
1st wave *N* = 165				0.18			0.16			0.26			0.30			0.24
Score on additive index on decentralized decision authority*	0	1	Reference		1	Reference		1	Reference		1	Reference		1	Reference	
	1	0.65	0.18; 2.34		0.69	0.18; 2.64		0.69	0.17; 2.79		0.72	0.18; 2.89		0.67	0.16; 2.82	
	2	1.54	0.49; 4.83		1.65	0.49; 5.58		1.61	0.44; 5.89		1.65	0.47; 5.86		1.57	0.42; 5.91	
	3	0.78	0.26; 2.38		0.78	0.24; 2.53		0.82	0.24; 2.76		0.80	0.25; 2.62		0.73	0.21; 2.53	
	4	0.69	0.18; 1.90		0.64	0.19; 2.22		0.68	0.18; 2.56		0.73	0.20; 2.64		0.70	0.19; 2.67	
	5	0.21	0.04; 1.05		0.21	0.04; 1.14		0.21	0.04; 1.28		0.22	0.04; 1.22		0.18	0.03; 1.14	
2nd wave *N* = 137				0.13			0.24			0.17			0.14			0.19
Score on additive index on decentralized decision authority*	0	1	Reference		1	Reference		1	Reference		1	Reference		1	Reference	
	1	0.89	0.17; 4.60		0.96	0.19; 4.79		0.92	0.17; 4.90		1.03	0.19; 5.66		1.16	0.19; 7.12	
	2	0.93	0.22; 3.85		1.01	0.25; 4.12		1.07	0.24; 4.78		1.05	0.23; 4.82		0.99	0.20; 4.86	
	3	0.57	0.14; 2.21		0.63	0.16; 2.43		0.61	0.15; 2.54		0.54	0.13; 2.30		0.49	0.11; 2.22	
	4	0.59	0.13; 2.58		0.71	0.16; 3.09		0.77	0.16; 3.66		0.72	0.14; 3.59		0.65	0.12; 3.47	
	5	0.20	0.05; 0.87		0.25	0.06; 1.07		0.25	0.06; 1.09		0.22	0.05; 0.97		0.18	0.05; 1.06	

*Based on answers about who can make decisions on: Prioritizing projects at the department, Collaborations with other departments at the hospital, Decisions regarding quality control, Significant changes in patient service, and Significant changes in departmental routines

^A^Model 1: Adjusted for hospital

^B^Model 2: Adjusted for hospital, managers’ job category, management training, and management tenure

^C^Model 3: Adjusted for hospital, managers’ job category, management training, management tenure, and span of control

^D^Model 4: Adjusted for hospital, managers’ job category, management training, management tenure, span of control, and acute tasks

^E^Tests if the result is different for each group

Table 3 shows the ordinal logistic regression analyses on decision authority measured as single items and sickness absence at the ward-level. The analyses are stratified by data wave and in four models adjusted for hospital, manager's job category, management training, management tenure, span of control, and acute tasks. The results indicate that the items ‘prioritizing projects at the department', ‘collaborations with other departments', ‘making decisions regarding quality control', ‘making significant changes in patient service' and ‘significant changes in the routines of the departments' were associated with lower risk of ward-level sickness absence. Across the models, only the item ‘making significant changes in patient service' for wave two had a statistically significant association with sickness absence at the ward-level.

**Table 3:** Results from multinomial logistic regression analysis of the association on the five items on decentralized decision authority and ward-level sickness absence

		Crude			Model 1^A^			Model 2^B^			Model 3^C^			Model 4^D^		
		OR	95%-CI	P^E^	OR	95%-CI	P^E^	OR	95%-CI	P^E^	OR	95%-CI	P^E^	OR	95%-CI	P^E^
1st wave	Item															
*N* = 165	1. Prioritizing projects at the department	0.83	0.46; 1.50	0.54	0.79	0.44; 1.41	0.42	0.77	0.43; 1.28	0.38	0.75	0.42; 1.34	0.32	0.75	0.42; 1.35	0.33
	2. Collaborations with other departments at the hospital	0.60	0.32; 1.13	0.12	0.61	0.33; 1.14	0.12	0.66	0.35; 1.26	0.21	0.65	0.34; 1.24	0.19	0.64	0.33; 1.22	0.17
	3. Decisions regarding quality control	0.78	0.39; 1.56	0.48	0.76	0.38; 1.52	0.43	0.81	0.30; 1.67	0.57	0.80	0.38; 1.67	0.56	0.89	0.42; 1.87	0.75
	4. Significant changes in patient service	0.50	0.23; 1.08	0.10	0.52	0.24; 1.11	0.11	0.51	0.23; 1.14	0.12	0.50	0.23; 1.11	0.11	0.52	0.24; 1.15	0.12
	5. Significant changes in departmental routines	0.88	0.46; 1.66	0.70	0.91	0.49; 1.70	0.77	0.92	0.48; 1.73	0.79	0.97	0.51; 1.87	0.96	0.97	0.50; 1.87	0.91
2nd wave	Item															
*N* = 137	1. Prioritizing projects at the department	0.77	0.40; 1.50	0.45	0.82	0.43; 1.56	0.54	0.77	0.39; 1.54	0.47	0.72	0.36; 1.44	0.36	0.70	0.33; 1.47	0.35
	2. Collaborations with other departments at the hospital	0.55	0.29; 1.04	0.07	0.63	0.34; 1.17	0.15	0.66	0.35; 1.22	0.19	0.69	0.37; 1.30	0.15	0.70	0.36; 1.36	0.29
	3. Decisions regarding quality control	0.55	0.29; 1.04	0.05	0.58	0.31; 1.10	0.07	0.66	0.34; 1.27	0.19	0.62	0.31; 1.23	0.15	0.61	0.30; 1.26	0.18
	4. Significant changes in patient service	0.30	0.13; 0.67	< 0.01	0.31	0.13; 0.73	< 0.01	0.30	0.13; 0.71	< 0.01	0.27	0.12; 0.65	< 0.01	0.27	0.12; 0.58	< 0.01
	5. Significant changes in departmental routines	0.87	0.45; 1.68	0.68	0.97	0.50; 1.88	0.92	0.95	0.48; 1.87	0.88	0.77	0.37; 1.70	0.70	0.60	0.32; 1.54	0.40

^A^Model 1: Adjusted for hospital

^B^Model 2: Adjusted for hospital, managers’ job category, management training, and management tenure

^C^Model 3: Adjusted for hospital, managers’ job category, management training, management tenure, and span of control

^D^Model 4: Adjusted for hospital, managers’ job category, management training, management tenure, span of control, and acute tasks

^E^P-value for OR-estimates

Table 4 shows the ordinal logistic regression model where ward-level sickness absence was stepwise adjusted for the relevant covariates. The results show that the higher the extent of cross-functional decision authority, the lower the odds of ward-level sickness absence. Adjusting for the covariates in Model 1–4 only change the risk estimates a little. The results are statistically significant for the first wave. The risk estimates from both waves and for all models show the same tendency.

**Table 4:** Results from the ordinal logistic regression analysis on the association between cross-functional decision authority and ward-level sickness absence

		Crude			Model 1^A^			Model 2^B^			Model 3^C^			Model 4^D^		
		OR	95%-CI	P^E^	OR	95%-CI	P^E^	OR	95%-CI	P^E^	OR	95%-CI	P^E^	OR	95%-CI	P^E^
1st wave				0.05			0.04			0.03			0.02			0.06
*N* = 163*	Not at all	1	Reference			Reference			Reference			Reference			Reference	
	To a lesser extent	0.98	0.32; 2.99		1.04	0.33; 3.27		0.97	0.31; 3.06		0.91	0.29; 2.92		0.96	0.29; 3.21	
	To some extent	1.07	0.36; 3.16		1.06	0.35; 3.19		1.03	0.35; 3.04		0.94	0.31; 2.86		1.03	0.33; 3.20	
	To a great extent	0.38	0.13; 1.17		0.38	0.12; 1.17		0.35	0.11; 1.07		0.32	0.11; 0.99		0.39	0.12; 1.26	
	To a very great extent	0.08	0.01; 0.49		0.08	0.01; 0.51		0.09	0.02; 0.55		0.08	0.01; 0.47		0.09	0.01; 0.56	
2nd wave				0.16			0.13			0.15			0.12			0.18
*N* = 137	Not at all	1	Reference		1	Reference		1	Reference		1	Reference		1	Reference	
	To a lesser extent	0.58	0.12; 2.79		0.64	0.18; 2.24		0.66	0.19; 2.34		0.69	0.18; 2.62		0.76	0.19; 2.99	
	To some extent	0.42	0.10; 1.84		0.43	0.13; 1.39		0.41	0.13; 1.30		0.41	0.12; 1.44		0.44	0.12; 1.60	
	To a great extent	0.24	0.05; 1.15		0.25	0.07; 0.93		0.25	0.07; 0.91		0.22	0.05; 0.89		0.25	0.06; 1.10	
	To a very great extent	0.17	0.02; 1.25		0.19	0.03; 1.25		0.19	0.03; 1.22		0.19	0.03; 1.25		0.20	0.03; 1.37	

^A^Model 1: Adjusted for hospital

^B^Model 2: Adjusted for hospital, managers’ job category, management training, and management tenure

^C^Model 3: Adjusted for hospital, managers’ job category, management training, management tenure, and span of control

^D^Model 4: Adjusted for hospital, managers’ job category, management training, management tenure, span of control, and acute tasks

^E^Tests if the result is different for each group

^*^ Information on cross-functional decision authority missing for 2 persons

## Discussion

### Principal Results and Comparison with Prior Work

The increase in care and work complexity in hospitals alongside growth in staff turnover and sickness absence requires appropriate and efficient management and decision authority at the right level in the organization [14, 15]. Our first hypothesis expected that decentralization of decision authority would result in lower levels of sickness absence in the wards. The absolute effect sizes shown in the odds ratios (for index-scores of 3, 4 and 5, albeit statistically non-significant) suggest that wards where frontline managers' report higher levels of decentralized decision-making authority have lower levels of registered sickness absence. Adjusting for relevant covariates (hospital, managers’ education, management training, tenure of the individual manager, span of control, and acute tasks) did not change the results. While our measure on decentralization of decision-making authority does not necessarily correlate with supportive leadership behaviours, it is likely that frontline managers with high levels of decision-making authority have better opportunities for exhibiting supportive leadership behaviours. A further explanation is that decision-making authority gives employees the experience of having greater work control, which according to previous studies reduce absence from work [53]. When studying the individual items that make up the composite measure of decentralized decision-making authority, the analyses only yielded few statistically significant associations. We found, however, that three of the five items in the measure exhibited higher correlations with the outcome measure. The self-reported ability to make decisions on ‘significant changes in patient services' had the strongest association with sickness absence at the ward-level, which is in line with other studies that find that the ability to focus on the core task in doing their job (taking care of patients) is important for employee well-being [54]. Our analysis also indicates that the possibility for frontline managers to make decisions on collaborations with other departments and quality control are determinants of workers well-being as measured by sickness absence.

Our second hypothesis expected that cross-functional decision authority would be associated with lower levels of sickness absence at the ward-level. In both waves of the study we observed tangible reductions in the odds ratios as the participants reported higher levels of cross-functional decision authority. Relatedly, the analysis suggests a similar pattern in both waves and when adjusting for factors such as hospital, manager’s job category, management training, tenure of the individual manager, span of control, and acute tasks. This indicates that frontline managers who hold a leadership role for a variety of professional groups in hospital settings may facilitate interdisciplinary completion of work tasks. This highlights that to facilitate the coordinated efforts of a multi-professional group of workers, managers must be more attentive towards bridging knowledge across boundaries and providing the necessary decision-making information, something previous studies have found to empower frontline workers [1, 55]. The stepwise adjustment for span of control in the statistical analyses had little impact on the observed associations between predictors and outcomes. This finding is noteworthy, because previous studies have argued that a high span of control is believed to challenge the basis for performing supervisory and consensual management [56].

Overall, the study findings indicate that decentralized management both directly and indirectly may constitute job resources that enhance the capacity of employees to deal with the work tasks while simultaneously supporting the well-being of employees [37]. The two aspects of decision authority may constitute job resources in their own right by enhancing the agility of the organization to deliver efficient and high-quality health care services. They may also indirectly serve as job resources by fostering supportive leadership behaviours, cross-functional cooperation, social support and job control that all have been found to be associated with lower levels of sickness absence [27]. It could be argued that higher levels of decentralized management could constitute an additional job demand on line managers negatively impacting their capacity to provide effective and supportive leadership, which again could entail an increase in the level of sickness absence. The study findings, however, do not support such an interpretation.

### Practical implications

By studying how frontline management influences the sickness absence in hospitals, our findings speak to the societal challenge of findings ways to reverse the projected shortage of healthcare staff in OECD countries [1, 4] that in part results from a poor work environment and conditions. For example, in Denmark, the hospital sector is among the work sectors with the highest prevalence of sickness absence (5.1% of the total working time). At the same time, care tasks are becoming increasingly complex processes because of shorter in-patient stays, patient input and process uncertainty, and the need for (a)synchronous work inputs from many healthcare professionals and organizational units [57]. As healthcare systems reorganize to meet those changes and in doing so face the risk of increasing sickness absence because of the uncertainty it brings [58, 59], our study provides additional backing for supporting frontline managers to manage in a way that fosters collaboration [44] and wellbeing at work [7] capable of developing a sustainable psychosocial work environment. The findings also highlight the importance of having good supervisor-employee relationships, which especially nurses associate with having adequate discretionary power to do their job [1]. By focusing on enhancing frontline managers’ decision authority in hospital settings and on improving other aspects of the psychosocial work environment, it should be possible to reduce sickness absence levels and, hence, improve work attendance of hospital employees.

### Study strengths and limitations

The results reported in this study are consistent across manager and hospital characteristics and across two independent survey waves in time at two Danish hospitals. To that end, a number of covariates were included in the analyses that might constitute potential confounders in the association between the independent and the dependent variable. This supports the credibility of the findings of the study. Compared to previous studies primarily surveying nurse managers, our study sample consists of nurses and physicians who jointly undertake the role of frontline managers, exemplifying a recent trend in clinical leadership models [25]. The study complements previous studies on hospital managers focusing on performance management [8] or specific leadership styles [12] such as distributed [14, 16] or relation-oriented leadership [5, 26]. Moreover, the study complements research on psychosocial working conditions (e.g., high workload and low levels of social capital, job control, and organizational justice) found to be predictors of sickness absence [27–31].

Future research should consider the following study limitations. The study has a relatively small sample size of frontline managers in the two waves (165 and 137 in the two hospitals), but it was tied to 121 wards with 3,680 employees in round one and 108 wards with 3,331 employees in round two, respectively. The sample size could also explain why only a few results in Tables 2, 3 and 4 are statistically significant. Yet, it is important to note that the sizes of the observed crude odds ratios are considerable, suggesting that the analyses are underpowered and that discarding the results due to statistical non-significance could lead to drawing ‘false-negative’ conclusions. The concept of decision authority was captured by five questionnaire items whereas the more experimental cross-functional decision authority was captured by only one questionnaire item could signal a call for a finer-grained understanding of the measures in future research studies. Because little quantitative research has measured cross-boundary decision authority, it is relevant to investigate further whether it is a structural mechanism that positively impacts healthcare workers’ well-being. Since sickness absence for the second wave of the survey was measured during 2020, the COVID-19 pandemic may have had an impact on the level of sickness absence in the participating wards. In Denmark, the COVID-19 lockdown was put into effect on 11 March, 2020. This must be taken into account when interpreting the results of the study, but because the results from the two waves (Table 2 and 3) show similar tendencies it suggests that the COVID-19 pandemic only had a limited impact on the results. It may also be considered a study limitation that the two waves of the study are not fully independent, since some participants in the first wave also participated in the second wave. To take this lack of independence into account in the analyses, we analysed the two waves separately.

## Conclusion

This two-wave, empirical study of frontline managers indicate that higher levels of decision authority and cross-functiona*l* decision authority in hospital wards are positively associated with lower risks of sickness absence. In this context, the study indicate that decentralized decision authority and cross-functional decision authority are important to the work environment in hospitals, and that the two management factors are capable of mitigating the challenges arising from hospital specialisation and task complexity. The study findings support conducting future research on how to empower healthcare professional’s decision autonomy at the frontline level of management in hospital wards.

### Supplementary Information

Additional file 1:

## Data Availability

The data used in this study are the property of the Capital Region of Denmark. Restrictions apply to the availability of these data, which were used under license for the current study, and are not publicly available. Data are, however, available from the authors upon reasonable request and with permission from the Capital Region of Denmark.
