# Supplementary material for: Impact of decentralized management on sickness absence in hospitals: a two-wave cohort study of frontline managers in Danish hospital wards
Source: BMC Health Serv Res. 2024 Jul 16;24:816. doi: 10.1186/s12913-024-11234-2 (PMC11250969; doi:10.1186/s12913-024-11234-2)
Supplement: Supplementary file 1 — Additional file 1: [file 12913_2024_11234_MOESM1_ESM.docx]

*Supplementary file 1*

**Impact of decentralized management on sickness absence in hospitals: a two-wave cohort study of frontline managers in Danish hospital wards**

Thim Prætorius*

Steno Diabetes Center Aarhus

Thomas Clausen, Ann Dyreborg Larsen, Jonas Kirchheiner-Rasmussen

National Research Center for the Working Environment

Lykke Margot Ricard. Peter Hasle

University of Southern Denmark

--------------------------------------------------------------------------------------------------

**Survey questions used in the article. Here translated into English from Danish.**

1. Sex

2. Profession

Response categories: Nurse; Chief physician; Junior doctor; Midwife; Physio/occupational therapist; Radiographer; Bioanalyst; Medical Secretary; Optician; Other (please write)

3. Year of birth

4. How many years have you been employed at the current hospital?

5. What year did you complete your primary education?

6. In which area of care are you a manager?

Response categories: Emergency department / Emergency room; Anesthesiology; Dermato-venereology; Endocrinology; Gastro/hepatology; Geriatrics; Gynecology and obstetrics; Hematology; Cardiology; Infection medicine; Vascular surgery; Surgical Gastroenterology; Surgery; Lung diseases; Nephrology; Neurosurgery; Neurology; Ophthalmology; Oncology; Orthopedic surgery; Paraclinical diagnostic; Plastic surgery; Pediatrics; Rheumatology; Thoracic surgery; Trauma reception; Urology; Ear-nose-throat; Other (please write)

7. How many years have you held your current position as manager?

8. How many years of experience do you have in total with management?

9. What formal management training have you completed?

Response categories (you may tick off multiple): Leadership program in the Capital Region of Denmark; External leadership program; Master's degree in management; Diploma in management; Other management training; No formal management training.

10. What type of care unit are you managing?

Response categories: Outpatient; Bed section; Surgery; Other (please write)

11. What type of task is handled at the unit you manage?

Response categories: Primarily elective; Primarily acute; Sub-acute; Both acute and elective; Other (please write)

12. Who has the decision-making authority with respect to:

a) prioritizing projects at the department, b) collaboration with other departments at the hospital, c) decisions with regard to quality control, d) significant changes in patient service, and e) significant changes in departmental routines.
Response categories: the decision authority lies with a) employees under my leadership, b) myself (and co-managers), c) my immediate supervisor, and d) the top management.

13. How many employees are you responsible for on a typical working day as unit manager?

14. To what extent do you on a daily basis exercise management for professional groups other than your own?

Measured on a 5-point Likert scale ranging from ‘not at all’ to ‘to a very great extent’
